# Too Good to be True? Ideomotor Theory from a Computational Perspective

**DOI:** 10.3389/fpsyg.2012.00494

**Published:** 2012-11-12

**Authors:** Oliver Herbort, Martin V. Butz

**Affiliations:** ^1^Department of Computer Science, Faculty of Science, Eberhard Karls Universität TübingenTübingen, Germany; ^2^Department of Psychology, Faculty of Science, Eberhard Karls Universität TübingenTübingen, Germany

**Keywords:** ideomotor theory, associative learning, computational model, planning, consolidation

## Abstract

In recent years, Ideomotor Theory has regained widespread attention and sparked the development of a number of theories on goal-directed behavior and learning. However, there are two issues with previous studies’ use of Ideomotor Theory. Although Ideomotor Theory is seen as very general, it is often studied in settings that are considerably more simplistic than most natural situations. Moreover, Ideomotor Theory’s claim that effect anticipations *directly* trigger actions and that action-effect learning is based on the formation of direct action-effect associations is hard to address empirically. We address these points from a computational perspective. A simple computational model of Ideomotor Theory was tested in tasks with different degrees of complexity. The model evaluation showed that Ideomotor Theory is a computationally feasible approach for understanding efficient action-effect learning for goal-directed behavior if the following preconditions are met: (1) The range of potential actions and effects has to be restricted. (2) Effects have to follow actions within a short time window. (3) Actions have to be simple and may not require sequencing. The first two preconditions also limit human performance and thus support Ideomotor Theory. The last precondition can be circumvented by extending the model with more complex, indirect action generation processes. In conclusion, we suggest that Ideomotor Theory offers a comprehensive framework to understand action-effect learning. However, we also suggest that additional processes may mediate the conversion of effect anticipations into actions in many situations.

## Introduction

Human beings are continuously confronted with change and novelty. Novel tools emerge, the environment changes, the social role of an individual changes, and the body grows and ages. Human beings can only deal with change and novelty because they can learn. *Ideomotor Theory* proposes a mechanism for learning to reach ones goals in novel situations. Ideomotor Theory is simple, old, elegant, and thus highly attractive (Herbart, 1825; Laycock, 1845; James, 1890; for a review of its history see Stock and Stock, 2004). It is a core element of many contemporary theories of goal-directed action (e.g., Hommel et al., 2001; Hoffmann, 2003) and has found considerable empirical support (e.g., Elsner and Hommel, 2001; Kunde et al., 2004, for a review see Shin et al., 2010). Its principles have also been picked up in other domains, such as social cognition (Paulus, 2012).

What follows is a brief summary of Ideomotor Theory. Whenever a movement is executed, the (mental representation of the) movement gets associated with (the mental representation of) its effects. This association between movement and effect is bidirectional. If the organism later wants to reach a goal state, the mere *anticipation* of this state may be sufficient to *directly* trigger the appropriate movement. This simple principle has been elaborated in more detailed theories of goal-directed action. For example, the theory of anticipatory behavior has put additional emphasis on the situation dependency of action-effect relationships (Hoffmann, 1993, 2003; Stock and Hoffmann, 2002). The Theory of Event Coding provides a sophisticated representational structure (Hommel et al., 2001).

Ideomotor Theory and many of its successors share three core assumptions. First, to trigger an action, the effects of the action are anticipated (effect anticipation). Second, this anticipatory image of action-effects directly activates an action by means of direct associations between actions and effects (direct-activation). Third, during learning these associations are acquired more or less independently of the actor’s current intentions and possibly without the help of a teacher (associative learning rule). This formulation of Ideomotor Theory, especially the direct-activation claim, distinguishes it from other approaches and can be called the “strong” Ideomotor Theory (Shin et al., 2010).

The effect anticipation assumption is supported by a range of experiments. A common feature of these experiments is that they show that the selection, initiation, and control of an action is affected by the features of its effects. An example is an experiment in which the response-effect-compatibility was manipulated (Kunde, 2001). In each trial, participants were asked to press one of four horizontally arranged keys in response to a non-spatial color stimulus. Each key press was followed by an effect stimulus in one of four horizontally arranged positions on a screen. If the positions of the keys corresponded to the positions of their effects, responses were faster than when there was no such spatial response-effect compatibility. Similar effects have been reported for other kinds of actions and stimuli, including social behavior (Kunde et al., 2004, 2011).

Likewise, the direct-activation claim has found empirical support. For example, electrophysiological and neuroimaging studies have shown that the mere perception of stimuli that were used as action-effects in an acquisition phase activated motor areas (Elsner et al., 2002; Melcher et al., 2008; Paulus et al., 2012). However, it remains unclear whether this activation results from direct action-effect links, as suggested by Ideomotor Theory, or if the link is mediated by other, potentially automatic, processes. It also remains to be studied if such observations can be confirmed for action-effect learning in more complex tasks.

Finally, it is hard to test the associative learning rule claim empirically. Even though action-effect learning shares characteristics with associative learning (Elsner and Hommel, 2004), it is difficult to draw conclusions about the underlying learning mechanisms. To conclude, Ideomotor Theory offers an astonishingly simple and elegant mechanism to explain the acquisition and execution of goal-directed actions. However, although the theory found empirical support, it is surprising that the assumed mechanisms have barely been adopted in computational models or machine learning algorithms.

From a psychological point of view, it is suspicious that Ideomotor Theory has rarely found a way into computational models of human learning and goal-directed actions. For example, in the domain of motor learning and control, only a few computational models can be considered direct implementations of Ideomotor Theory (e.g., Herbort et al., 2005). Most approaches differ considerably (for reviews see Wolpert et al., 2001; Todorov, 2004; Butz et al., 2008).

From a functional point of view, it can be argued that Ideomotor Theory has mostly been studied in rather simple settings. In experiments the range of relevant actions and effects is constrained, the to be executed actions are usually simple, and the effects quickly follow actions. While these features are shared by some real-world learning tasks, many real-world situations have less clearly identifiable action and effect dimensions, require the execution of more complex actions, and provide delayed effects only. Thus, even though recent experiments progressed toward studying action-effect learning in more realistic settings (e.g., Paulus et al., 2012), it remains unclear to what extent Ideomotor Theory is applicable to more complex learning tasks. Doubt of the applicability of Ideomotor Theory in such situations is also raised by the fact that many machine learning techniques and artificial intelligence approaches have little in common with Ideomotor Theory.

The previous considerations show that Ideomotor Theory is a well-accepted framework. Nonetheless, there are reasons to question whether the theory fully lives up to its claims. Here, we adopt a computational perspective to put Ideomotor Theory to the test. To this aim, we cast Ideomotor Theory in a simple computational model that is based on the theories’ basic claims. We then evaluate the performance of the model in a series of tasks to test if it reproduces empirical findings. Our goal is to test Ideomotor Theory with our model, rather than developing a model that strives to account all behavioral findings related to action-effect learning. Each task aims to capture the essence of a real-world challenge for any learning mechanism. During our exploration, we take two different perspectives. The psychological perspective considers whether Ideomotor Theory parallels human behavior, in both success and failure. The functional perspective considers which kinds of tasks can be mastered with Ideomotor Theory. This includes the question of whether it can account theoretically for learning to coordinate actions in tasks other than those previously studied in the lab.

Evidently, the brain relies on other learning mechanisms besides the one specified by Ideomotor Theory (Doya, 1999). Consequently, the failure or success of our model in specific tasks could be attributed to mechanisms other than the one proposed by Ideomotor Theory. Nevertheless, an isolated computational analysis of Ideomotor Theory will shed additional light on its validity. First, a computational analysis of various learning tasks allows us to test whether Ideomotor Theory specifies a basic learning mechanism that bootstraps the acquisition of goal-directed behavior. Second, even though other learning mechanisms may complement Ideomotor Theory, it is important to know how far one can go with Ideomotor Theory alone and under which conditions Ideomotor Theory fails or requires complementary mechanisms. Finally, a computational analysis of various learning tasks may help to identify potential challenges for Ideomotor Theory.

## A Computational Model of Strong Ideomotor Theory

**Figure 1:**
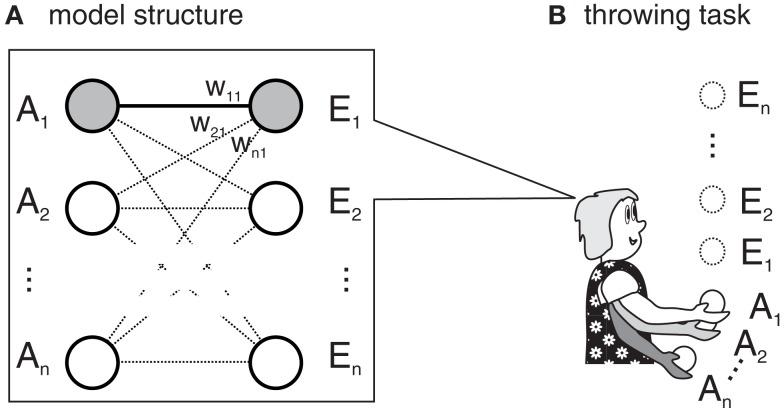
**(A)** According to Ideomotor Theory, actions (*A*_1_,…) get associated to their effects (*E*_1_, …) when a person learns a novel task, such as throwing a ball. **(B)** In the example, each action node activates a throw of different strength and each effect node encodes the height of the resulting ball flight. If *A*_1_ resulted in *E*_1_ (gray circles), the link between both (*w*_11_) is strengthened.

In this section, we outline a simple computational model which strives to capture the core ideas of Ideomotor Theory without adding unnecessary features. Figure 1A depicts the general layout of the model. It is comprised of a simple, single-layered neural network containing two sets of nodes: action nodes and effect nodes (*A*_1_, *A*_2_, …, *A*_n_, and *E*_1_, *E*_2_, …, *E_n_*). For each action, there is one action node and for each effect, there is one effect node. If an action is executed or an effect is perceived, the respective nodes are active (i.e., activity is set to 1.0). If no action is executed or no effect is perceived, they are mute (activity is set to 0.0). The action-effect association *w_ij_* between an action node *A_i_* and an effect node *E_j_* is strengthened when both nodes are active at the same time.

We apply this mechanism to a simple exemplar task: learning to throw a ball to particular heights. Each action node is associated to a throwing movement with a particular strength. In our example, activating *A*_1_ causes a weak throw, activating *A*_2_ causes a somewhat stronger throw, and so on. In this view, an action is defined as the production of a throw with specific strength, and throws of different strengths are considered different actions. Each effect node encodes a specific height. During learning, actions are randomly and individually executed^1^. Thus, there is only one active action node which gets associated to the activated effect nodes. In our model, the weight of the association between two active nodes is increased by one. To produce goal-directed behavior an effect node is activated and the activity is spread to the action nodes. If the goal is to produce the effect associated with the *i*-th effect node, each action node *A_j_* is activated by the value *w_ji_*. To select an action node, a winner-takes-all procedure is applied by selecting the action node with the highest activation. If there are several nodes with maximal activation, one of them is selected randomly. This formulation is fairly simple, but it captures Ideomotor Theory’s three main assumptions: effect anticipation, direct-activation, and the associative learning rule.

### Model features

#### Learning rule

Although we kept the model as simple and generic as possible, we want to explain some design decisions before proceeding. First, artificial neural networks are usually modeled with non-linear nodes (e.g., node activations are restricted to a range from 0.0 to 1.0 by a non-linear, sigmoidal input function) or include mechanisms to bind the associative strength between two nodes. Because we select the action node with the highest activation in a winner-takes-all mode, such algorithms would not affect the predictions of our model in the tasks we employ.

#### Situation and context

Obviously, Ideomotor Theory as it is formulated above is an oversimplification because it does not take into account that actions may have different effects in different situations. The model could be easily extended to encode action-effect associations situation-dependently. To keep our model simple, we do not account for the situation from the beginning but will introduce situation-dependencies later.

#### Representation

For the sake of the simplicity of our model, we consider only a single action dimension and a single stimulus dimension. Of course, it would be possible to integrate more than one stimulus dimension. Indeed, it has been suggested that a population-code like representational structure, as is employed in our model, is especially suited to allow the integration of multiple stimulus dimension (Ma et al., 2006). Moreover, in our model the representational structure does not change. Each node consistently encodes the same action or stimulus. Thus, the model does not implement any mechanisms for changing the receptive field of the present nodes or introducing new nodes. These simplifications are justified for three reasons. First, our tasks can be learned without adaptations of the representational structure. Second, adaptation is only possible once some skill is acquired in a given task. Because we also want to test the claim that Ideomotor Theory can bootstrap learning, we exclude such mechanisms. Third, no such processes are specified by Ideomotor Theory and we aim to provide a proof of principle of Ideomotor Theory. Nevertheless, future modeling might greatly benefit from integrating Ideomotor Theory with a richer, adaptive representational structure.

### Task

As a simple scenario for our model evaluation, we refer to the example of a child that is about to learn to throw a ball to various heights. In the example, actions are defined as throws of different strengths. The child can also perceive the position of the ball (Figure 1B). While we keep the task as simple as described here in the first test case, it is subsequently enriched. The task will be changed with respect to the action-effect mappings and the dynamics of actions and effects. However, some aspects of the task will stay constant. First, learning is always unsupervised. This means that the model receives neither reinforcement signals (such as “this action was good”) nor corrective feedback (such as “next time better use action X”) from an external teacher or from internal prior knowledge. This reflects the central claim of Ideomotor Theory that goal-directed actions can be acquired solely by observing the effects of own movements. Second, the same associative learning rule will be applied in all settings. Third, the representational structure will remain fairly constant, with the exception being that the number of action and effect nodes will be varied.

### Evaluation

The model can be tested by selecting a goal state and activating the associated effect node. The action then suggested by the model can be read out as described above. If the action produces the desired effect, it can be considered a success. To evaluate the performance of the model in various tasks, we generate a number of independent instances of the model and train them. At various time points during training we require each instance of the model to reach each possible goal state. If the model outcome is stochastic, each goal is presented repeatedly. As a measure of performance, we report the percentage of successful actions, averaged over all goals, repetitions, and model instances. Later, we distinguish between successful and optimal actions. Optimal actions are defined as successful actions that produce the goal in the most efficient way. When a model is tested, no novel action-effect associations are formed.

### Roadmap

In the following, we present five different scenarios in which we examine the performance of Ideomotor Theory in the face of different challenges imposed by many learning tasks. In Case 1, we show that the model is able to learn to control a task defined by a simple one-to-one mapping. Even if the number of actions or effects increases or noise is added, the model remains effective. In Case 2, we show that model performance degrades if multiple and potentially irrelevant actions can be executed in parallel. This implies that Ideomotor Theory explains learning best in a task in which actions and effects are clearly defined. In Case 3, we show that the model is able to encode redundant action possibilities, which is a central problem in motor learning. In Case 4, we extend the model by allowing actions to trigger a chain of effects at various time points. This case shows that learning in our model depends critically on the close temporal proximity between action and effect. Finally, in Case 5, we examine the scenario that a sequence of actions is necessary to produce an effect. It is shown that Ideomotor Theory has difficulties in learning longer action sequences. It is suggested that this shortcoming can be overcome by introducing additional mechanisms which, however, go beyond some of the core assumptions of Ideomotor Theory.

## Model Evaluation

### Case 1: One-to-one mapping between actions and effects

**Figure 2:**
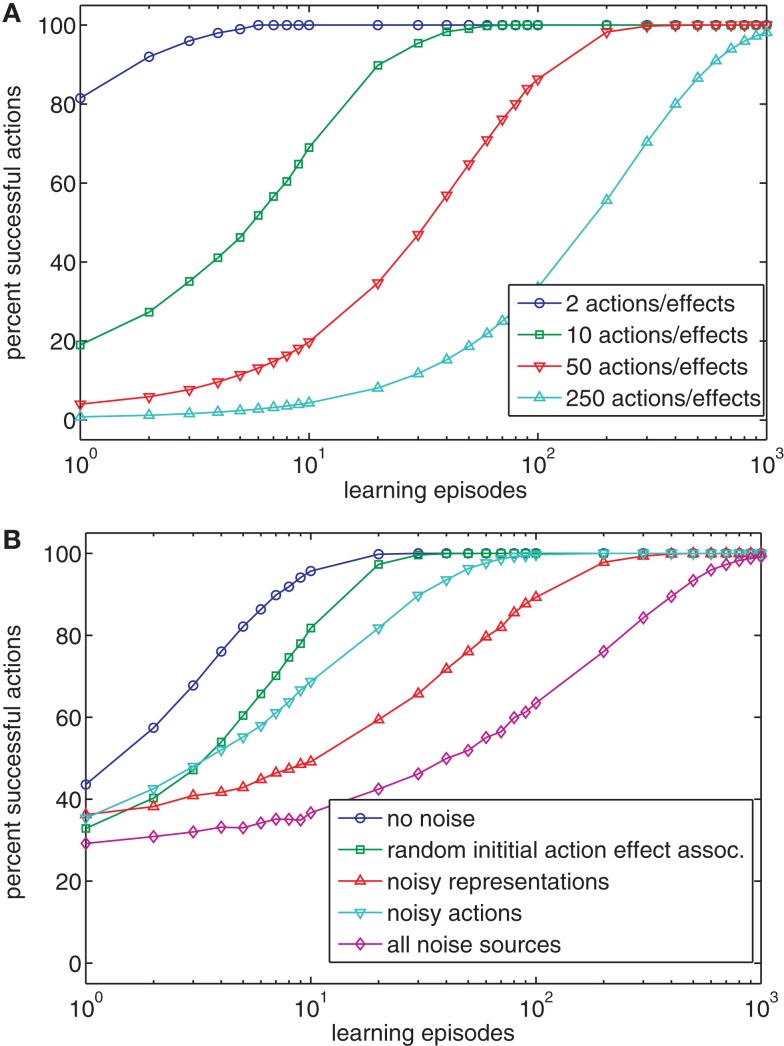
**(A)** The chart shows the percentage of successful actions after various numbers of learning episodes for scenarios with different numbers of possible actions and effects. **(B)** The chart shows the impact of various kinds of noise on the performance of the model of Ideomotor Theory (using four actions/effects).

The simplest learning task is that of a one-to-one mapping between actions and effects. In this case, each action produces one specific effect and each effect is produced by one specific action. This case closely describes many experiments on Ideomotor Theory in which participants usually perform clearly defined actions (e.g., button presses) that are accompanied by clearly defined effects (tones, e.g., Elsner and Hommel, 2001). It seems obvious that Ideomotor Theory can account for learning when only few different actions and effects are involved. In many situations, however, a much greater number of actions and effects are possible, and as a result we tested our model with different numbers of actions and effects (2, 10, 50, 250). Figure 2A shows the results of 100 simulated runs for each number of actions and effects. If the number of possible actions and effects is low (e.g., 2 or 10), the model of Ideomotor Theory is able to produce different effects after very few trials. This corresponds to the results of Wolfensteller and Ruge (2011), who report action-effect learning after very few repetitions of different possible action-effect episodes. However, if the number of distinguishable actions and effects increases, learning takes longer but still results in a high success rate. The main reason that learning slows down with a growing number of actions is that for maximal performance, each action has to be executed at least once. In sum, Ideomotor Theory can successfully account for goal-directed behavior in one-to-one scenarios.

As a first step toward more realistic situations we wanted to test whether learning is robust to noise. To do this we ran the simulation with four actions and effects and added noise during learning. To compare the conditions, noise was switched off during testing. In the no noise condition, we did not include noise. In one condition, we set the initial action-effect association weights to Gaussian distributed random values (*m* = 0.0, *sd* = 1.0). In another condition, we added random Gaussian noise to each node in each learning episode (*m* = 0.0, *sd* = 1.0). This corresponds to a situation in which neither actions nor effects can be encoded noise-free by the neural apparatus. In a third condition, the selected action was replaced by one of the other actions in half of all learning episodes. This corresponds to a clumsy child with a very noisy motor system^2^. Finally, we combined all noise conditions. Each condition was simulated 1000 times. Figure 2B shows that even though noise slows down learning, the behavior is successful in the end. Comparing the simulation data with empirical results suggests that action-effect learning is subject to very little noise in common experimental setups (Wolfensteller and Ruge, 2011). This seems reasonable, as actions and stimuli are usually easily distinguishable in the lab.

To conclude, the model accounts for action-effect learning in a simple task. If noise is low and the number of different actions and effects corresponds to the number used in experimental setups, the model requires about the same amount of training as humans do. When the number of potential actions and effects is high or when noise is present, learning is slower but still effective in the end.

### Case 2: One-to-one mapping with irrelevant actions

In the previous case, the child throwing the ball could only execute actions that were directly related to the task. However, while throwing, the child could have reoriented the head and the eyes, tapped with a foot, swayed the body, or could have talked. Thus, besides actions that have an immediate impact on the effect, many other actions can be executed in parallel. Consequently, the effects of the novel task may get associated to other, irrelevant actions.

**Figure 3:**
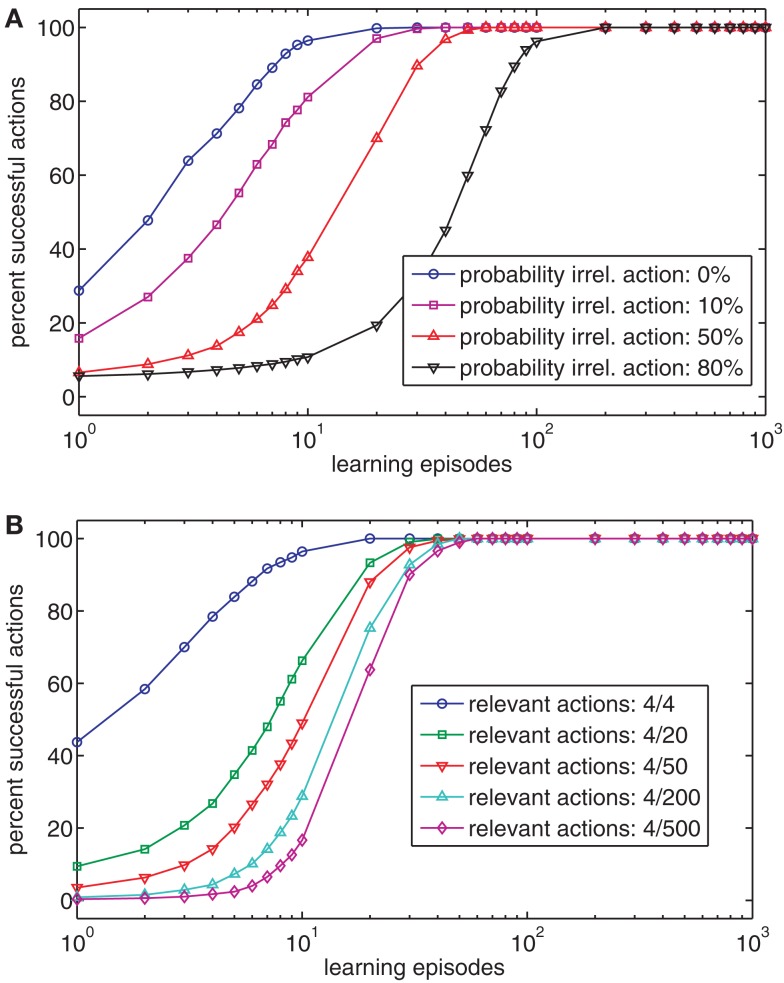
**(A)** The chart shows the effect of the relative frequency of activating irrelevant action nodes on ideomotor learning. **(B)** The chart shows the effect of the ratio of relevant and irrelevant actions on ideomotor learning.

To test whether this poses a problem, we added 16 irrelevant action nodes to the four relevant nodes in our model. The activation of irrelevant action nodes did not yield any effects (at least in the effect nodes under consideration). Each of the irrelevant action nodes was activated randomly with a fixed probability during training. In addition, one actually relevant action node was activated in every learning episode. Figure 3A shows that learning slows down with increasing probability of irrelevant action nodes being active. Thus, even a moderate ratio of relevant to irrelevant actions could decrease the speed of learning by up to an order of magnitude. Figure 3B also shows that the ratio of relevant to irrelevant action nodes affects initial learning, even though a high performance level is reached after some time. In the analysis, irrelevant action nodes were activated with a probability of 0.25.

It seems reasonable to assume that in many situations and tasks, the ratio of task-relevant actions to task-irrelevant actions is much less favorable than assumed in our (noise-free) examples. Thus, on its own, Ideomotor Theory provides a rather slow and ineffective learning mechanism. We see three ways to deal with this limitation. First, it can be acknowledged that learning a novel task without a teacher takes time. We discuss this issue in more detail in the general discussion. Second, one could assume that an attentional mechanism constrains the range of possibly to-be-associated action and effect nodes. However, from a learning perspective, this assumption is problematic. It implies that a more fundamental learning mechanism than that proposed by Ideomotor Theory pre-structures the learning problem and that Ideomotor Theory is insufficient for bootstraping learning. Third, the sparse coding scheme results in a high number of different action and effect representations. Nodes with broad receptive fields might be employed to first home in on the relevant action and stimulus dimensions of a task. The resulting constrained space of task-relevant action-effect might then be subject to action-effect learning as described in our model. Indeed, it has been shown that executing actions primes stimulus dimensions that relate to this action (Fagioli et al., 2007), and that infants turn attention toward relevant stimulus dimensions when skills improve (Eppler, 1995). However, even if more sophisticated representational structures might facilitate learning in our model, it must be kept in mind that most tasks are also much more complex than our exemplar one.

To conclude, Case 1 and Case 2 have shown that if the number of relevant actions and effect nodes is high and task-irrelevant actions can be executed during learning, the learning mechanism underlying Ideomotor Theory may be rather inefficient, even though it leads to an effective action selection in the end.

### Case 3: Redundant action possibilities

**Figure 4:**
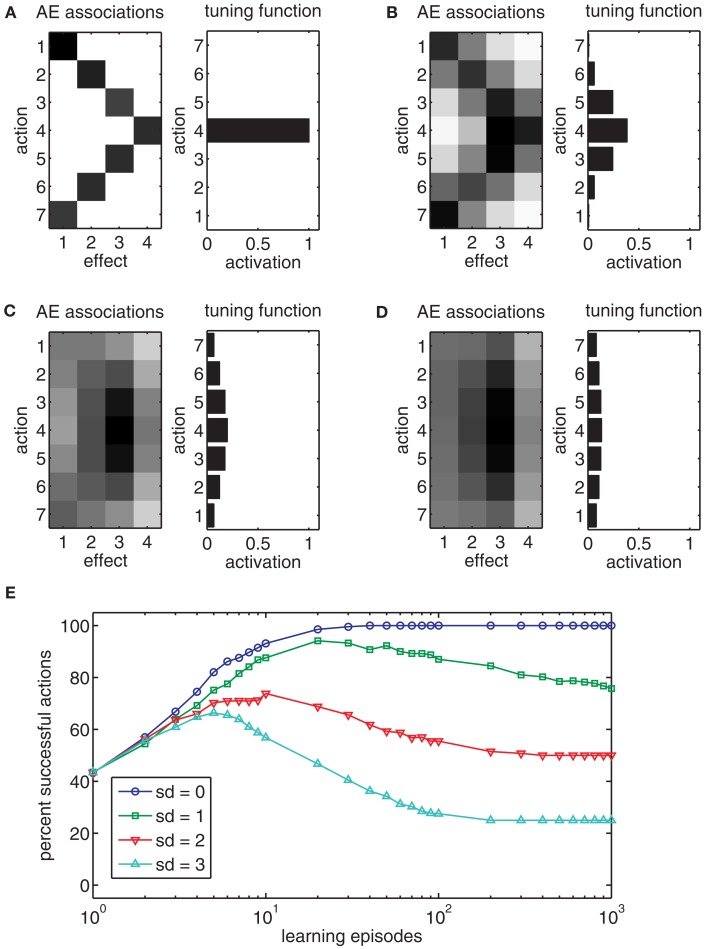
**(A–D)** The left chart of each panel shows the action-effect (AE) associations after 1000 episodes (darker squares indicate stronger associations) with different Gaussian action turning functions (SD = 0.0, 1.0, 2.0, 3.0). The right chart of each panel shows the exemplar tuning functions for action 4. **(E)** The chart shows the effect of the action tuning function on the performance of the model.

Up until now we have considered cases with one-to-one mappings between actions and effects. However, most goals can be reached in numerous ways. To accommodate this, the ball-throwing example is modified. Consider that the child is now tossing a paper plane and not a ball. To make the paper plane fly as far as possible, just the right amount of force is needed. This means that some flying distances (effects) can be reached either with a strong or a mild throw. To include this into our model we extended the range of actions. For the milder throws (*A*_1_–*A*_4_), increasing throwing force result in increasing flying distances (*E*_1_–*E*_4_). However, for the stronger throws (*A*_5_–*A*_7_), increasing throwing force results in decreasing flying distance (*E*_3_–*E*_1_). Humans face similarly structured situations all the time. For example, a specific hand position in 3D space can be realized by an infinite number of arm postures. Likewise, most objects can be grasped in different ways. Figure 4A shows that two distinct action nodes get associated to each of the effects *E*_1_–*E*_3_ during learning. For example, it is encoded that *E*_1_ can be realized by either executing *A*_1_ or *A*_7_. Thus, Ideomotor Theory is able to encode redundant action possibilities for each action.

This feature is not trivial, because many learning mechanisms (e.g., direct inverse modeling) can barely cope with similar problems (Jordan and Wolpert, 1999). The reason for this is that they cannot encode two or more distinct actions that result in the same effect. If several actions produce the same effect, these actions are blended into a single representation. Considering our example, a short flying distance would be associated with a mixture of weak and strong throws. Thus, a medium force throw would effectively be activated when striving for short flying distances, even if it effectively produces rather large flying distances. This problem is also referred to as the non-convexity problem (Jordan and Wolpert, 1999).

Even though Ideomotor Theory is not subject to the non-convexity problem under ideal conditions, its performance may degrade under more realistic circumstances. In the example of Figure 1A, the action nodes were tuned very sharply to specific actions, resulting in the activation of a single node. This precise representation results in the likewise accurate representation of the action-effect structure of the task. However, in neural systems, nodes are frequently tuned much more broadly (Georgopoulos et al., 1983; Bastian et al., 2003). To implement this finding, an action is now encoded by all action nodes based on a Gaussian tuning function, where the *i*-th action activates each node *A_j_* based on a Gaussian function with mean *i*^3^. Hence, when the *i*-th action is executed, not only is action node A_i_ active, but adjacent nodes are also active, albeit somewhat less so. To assess the effect of the breadth of the tuning function, we set its standard deviation to either 0, 1, 2, or 3. Figures 4A–D shows that the representation of redundant actions degrades with broader tuning curves. As a result, the model loses its ability to reproduce certain effects (Figure 4E). To conclude, Ideomotor Theory can be applied to some extent to redundant tasks if the tuning functions of actions nodes are sharp. Under more realistic conditions performance partially degrades.

### Case 4: Dynamic action-effects

In the previous cases, the potential delay between actions and effects was not considered. However, timing is an important factor in action-effect learning (Elsner and Hommel, 2004; Haering and Kiesel, 2012). Moreover, in the real word, effects are not only delayed, but less clearly defined than in the lab. For example, throwing a ball results in the ball passing through a number of states on the way to the peak of the trajectory and then down again. Likewise, the activation of muscles causes the body to transition through a number of states.

To test whether Ideomotor Theory can explain learning in such tasks, we made our scenario more dynamic. Upon the execution of one of four actions, the ball moves up and down on a parabolic path. The trajectory of the ball was modeled so that the strongest throw propels the ball to the peak of its trajectory within 0.5 s. The ball then falls down again for 0.5 s^4^. Depending on the rate with which the child updates the position of the ball, each action causes a number of successive effects. To be able to associate an action to these effects, we include a trace conditioning mechanism in the model (Pavlov, 1927). Each action node remains active after action execution for a certain time interval. Thus, it can be associated to delayed action-effects. While such a mechanism seems to be a prerequisite for learning, the learning task gets considerably more difficult because the action-effect relation is less clear-cut than in the previous cases.

**Figure 5:**
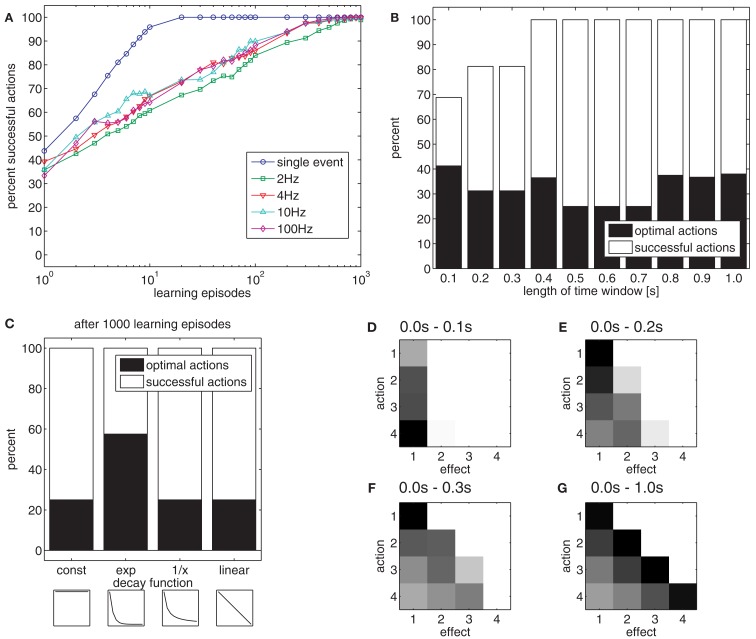
**(A)** The chart shows the effect of the different sampling rates on learning if effects unfold dynamically in time. **(B)** The chart shows the effect of time windows of different length on the acquisition of an effective (white) and optimal (black) action-effect mapping. **(C)** The chart shows the effect of different trace decay functions. The insets show the decay functions between 0.0 and 1.0 s after action execution: const: constant value in time window 0.0–1.0 s; exp, exponential decay; 1/x, inverse proportional; linear, linear decrease. **(D–G)** The charts show the strengths of action-effect associations after learning of 1000 episodes with a time window of 0.1 s **(D)**, 0.2 s **(E)**, 0.3 s **(F)** and 1.0 s **(G)**. Black squares denote strong connections between actions and effects, white squares denote no connections.

As a first step, we explored the sampling rate with which the effects are perceived. We used sampling rates of 2, 4, 10, and 100 Hz, meaning that the effect nodes are updated every 500, 250, 100, or 10 ms, respectively. Additionally, we included a condition in which the peak height of the ball was presented as a single effect. Under all conditions with dynamic effects, it was more difficult to learn to reproduce the different possible peak heights than under the single effect condition (Figure 5A).

These results suggest that a mechanism to single out the relevant event is crucial. In our example, this was difficult because the model was perceptually unable to distinguish between a ball on its way up, down, or at the peak of the trajectory. Indeed, from the perspective of the model, actions were mostly successful. As the model cannot perceive whether the ball is at its peak (e.g., has zero velocity) or not, it is sufficient – from the model’s perspective – to make the ball pass through a specific height to reproduce the respective effect. Indeed, when considering this, the model is highly accurate. This was easy, however, because most actions reproduce several effects. For example, all actions are suitable to make the ball travel through the lowest position *E*_1_.

#### Optimal actions

When many actions are suitable to reach a goal, one might ask which action should be selected. From a functional point of view, it is reasonable to select the most efficient action (Todorov, 2004). Because energetic costs and uncertainties are not included in our model, the most efficient or optimal action can be considered the action that produces an effect as quickly as possible. In our example, the optimal action is always the strongest throw (*A*_4_), because this action propels the ball to all possible positions faster than any other action. However, when the model associates an action to everything that happens later, suboptimal actions are chosen in 75% of cases (Figure 5B, right black bar).

To improve efficiency, one could assume that actions are only associated to those effects that occur within a short time window after action execution. Experimental results suggest that this time window spans between 1 and 2 s (Elsner and Hommel, 2004). Figure 5B shows the percentage of successful and optimal actions for different time windows, using a sample rate of 10 Hz. For short time windows, successful actions are more frequently optimal than for longer time windows. Nevertheless, the model cannot always generate successful actions if the time window is short. The reason for this is illustrated in Figures 5D–G. The Figures show the strength of action-effect associations acquired with time windows of different lengths. If the time window is short (0.0–0.1 s, Figure 5D), the most effective action (*A*_4_) is associated to *E*_1_, but because the ball needs more than 0.1 s to move into the receptive field of nodes *E*_2_–*E*_4_, these effects never get associated to any action. If the time window is longer, all effect nodes get associated with action nodes. However, widening the time window removes the bias to associate effects with those actions that produce the effect quickly, yielding inefficient action choices.

To assess whether this trade-off can be avoided by a more sophisticated trace decay function, different decay functions were compared. Figure 5C shows the usage of different decay functions, which modulate the strength for temporally distant action-effect associations. Whereas an exponential decay function yielded the best result, a linear and an inverse proportional decay function were just as inefficient as a constant function.

To conclude, we applied a variety of sample rates, time windows in which actions and effects would be associated, and trace decay functions in the learning tasks with dynamic effects. Except for the shortest time windows, most goals could be reached but action selection was rather suboptimal. If the time window was short or an exponential decay function was applied, optimal actions were selected more frequently. Although this poses a functional limitation, it corresponds to human action-effect learning (Elsner and Hommel, 2004). Thus, from a psychological perspective, this property of the associative learning rule supports the model of Ideomotor Theory. Therefore, Ideomotor Theory is also supported as an account for action-effect learning.

### Case 5: Stimulus dependency and sequential actions

In the previous cases the activation of a single action node resulted in some effects. However, many situations are more complex. Not only do action-effects depend on the current state of the body or the environment, but some effects may only be produced under conditions that need to be approached beforehand. For example, before a lifting movement of the arm causes the ball to fly, the ball needs to be grasped. In humans, even simple actions such as grasping a cup require the coordination of multiple movements (Herbort and Butz, 2011). Moreover, a concerted pattern of control signals needs to be sequenced to enable even simple arm movements (Gottlieb, 1996).

#### Direct and indirect (state-) action-effect associations

To test whether Ideomotor Theory is capable of sequencing actions, we altered our example in several ways. Consider for now that there are four actions, *A*_1_–*A*_4_. Each action moves the arm to a specific position. Action *A*_1_ moves the arm to a low position, action *A*_2_ to a higher position, and so on. We assume that the ball rests in the open hand. As long as the hand moves down or up slowly, the ball stays in the hand. In these cases, actions *A*_1_ to *A*_4_ result in effects *E*_1_ to *E*_4_, respectively. If the hand moves up quickly, the ball is thrown. The peak position of the ball trajectory depends on how far the hand has moved in the last step. If the hand starts from the lowest position, which is encoded as *E*_1_, and *A*_3_ is executed, the effect will be *E*_5_. If *A*_4_ is executed, *E*_6_ will result. In this example, many effects can be reached by simply executing a single action. However, in some cases several actions need to be sequenced. For example, if the ball should be thrown as high as possible (*E*_6_), one has to execute *A*_1_, to move the arm and ball down, and then *A*_4_, to propel the ball quickly upward. Likewise, if one has just generated *E*_1_ and now wants to produce *E*_4_, the arm needs to be brought up slowly by executing *A*_2_, *A*_3_, and then *A*_4_ in succession_._ If action *A*_4_ would be directly executed, the ball would be thrown in the air and *E*_4_ would not be reached^5^.

To be able to apply Ideomotor Theory to this example, it is necessary to make action-effect associations conditional on the current state. This conditionality is realized by adding a state layer. Kiesel and Hoffmann (2004) have provided empirical support for the state-conditionality of action-effect associations.

The state layer encodes the effect of the previous action and is otherwise functionally and structurally similar to the effect layer. State-action-effect associations are formed between active nodes of the three layers during learning, dependent on the state before the action, the action, and its effect. Unlike in the previous cases, a learning episode is now defined as a sequence of actions that lasts until the child produces a particular effect, which has been randomly determined before the episode. For goal-directed actions, the weights of those state-action-effect associations that match the current state and the desired effect are compared, and the action of the strongest state-action-effect association is executed.

**Figure 6:**
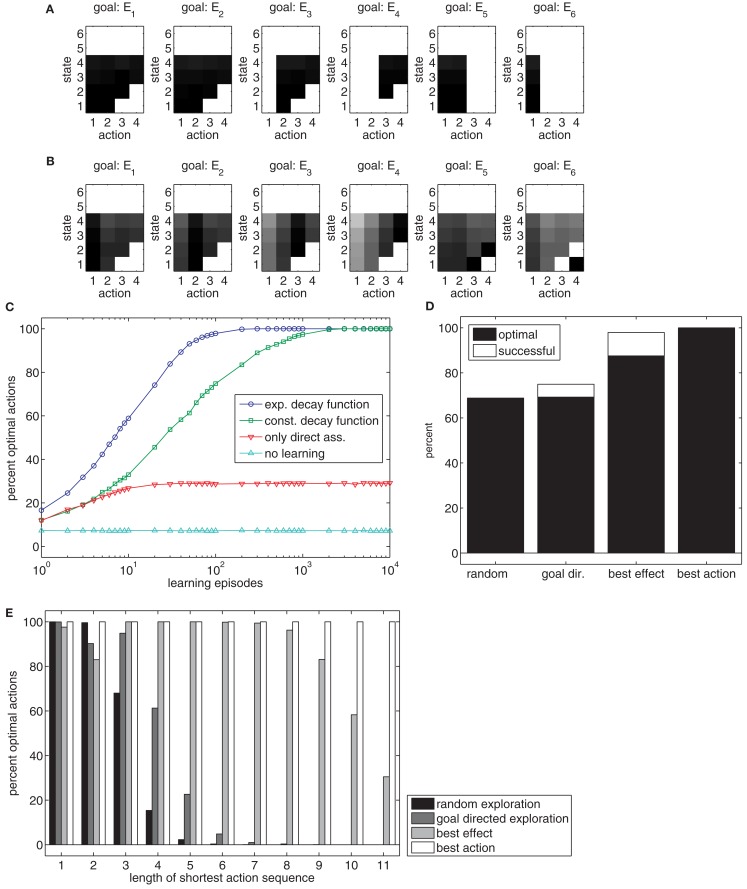
**(A)** The chart shows the weights of state-action-effect associations after 10,000 learning episodes when acquired without a trace of past actions. Black squares denote large weights, white squares denote no associations. **(B)** The chart shows the weights’ state-action-effect associations after 10,000 learning episodes when acquired with a trace of past actions. Please note, no actions are associated to states five and six because these are states in which the ball has already been thrown. **(C)** The chart shows how frequently goals are reached optimally (i.e., with a minimal actions sequence) with different trace decay functions. **(D)** The chart shows how efficient goals that require sequencing of at least two actions can be reached dependent on the learning method in a setup with 12 action nodes. **(E)** The chart shows how efficient goals can be reached, dependent on the minimal number of actions that need to be sequenced to reach the goal and dependent on the learning method.

When applying this model directly to the task, state-action-effect associations similar to those in Figure 6A are formed. An inspection of the chart reveals that several associations have been formed. However, some combinations of states and goals are not associated to any action nodes. For example, for goal *E*_6_ no action is associated with the states 1, 2, and 3. This lack of associations is due to the fact that some goals just cannot be reached *directly* from some states, but only by sequencing several actions.

Hence, Ideomotor Theory needs to be extended in a way that enables such sequencing. To allow this, a trace conditioning procedure similar to that of Case 4 can be used. If each state-action pair is not only associated to its immediate, direct effects, but also, due to its trace, to subsequent, indirect effects – just as is done to enable learning in the previous case – a sufficient structure of state-action-effect associations might be built. Figure 6B shows the state-action-effect associations for a model in which the executed states and actions were associated to all subsequent effects, using an exponential trace decay function. For each state in which the ball is still in the hand (1–4) and for each goal, at least one action can be derived.

#### Trace decay

To test the importance of different ways to associate later effects with state-action pairs, different decay functions were evaluated. Figure 6C shows performance curves for different learning methods with respect to combinations of initial states and goals which require sequencing actions (average of 100 simulations for each condition). An exponential decay function yielded the best results (blue circles)^6^. In contrast, learning is considerably slower if no discounting function is used (green squares). If only direct state-action-effect associations are formed, performance is heavily impaired (red triangles) but still outperforms a baseline condition without any learning in which random actions were chosen (light blue triangles). The difference between the latter two conditions arises because there is some chance that random actions result in a state from which the goal can be directly reached in the direct association condition, but not in the baseline condition.

This example shows that Ideomotor Theory is theoretically able to account for action sequencing. However, the example of four different actions is fairly simple. To evaluate whether learning is still possible in a more complex scenario, we scaled the example up to 12 possible actions (100 simulation runs). Figure 6D (left bar) shows that the goal is reached in only about two of three cases, even after 10.000 learning episodes. Further analysis reveals that if a goal can be reached by a single action or very short sequences of actions, the model produces almost optimal behavior. However, if three or more actions need to be sequenced, behavior fails almost all of the time (Figure 6E, black bars).

#### Action selection during learning

This leads to the question of why it is so hard to generate longer action sequences. There are at least two potential reasons. First, it is possible that the structure of our model lacks the power to store the information that is necessary to sequence actions. Second, it is possible that the model is not able to extract the information from the training data. In the following, we argue that the latter aspect limits the performance of the model. In our example, learning is based on the random execution of actions. In the previous cases this did not pose any problem, because each action was more or less useful to generate some effects. This has changed in the current task – while some action sequences are useful to produce an effect, others are not. Moreover, the probability that a long, useful action sequence is produced by chance drops exponentially toward zero with growing sequence length. However, the model needs to experience a long useful action sequence at least once to be able to reproduce it.

Thus, one can now ask how critical performance depends on the actions that are executed during learning. To test this, we implemented three additional methods for action generation during learning and trained the 12 action node model for 10.000 episodes, using an exponential trace decay function (100 simulation runs for each condition)^7^. These methods only affect the action choices; the learning mechanism for generating state-action-effect associations remains identical in all cases.

The *random exploration* method, which was used so-far, produced a new random action in each time step. Thus the child in the example just moves the arm to various positions, without trying to throw the ball in a specific way. As shown above, only mediocre results are achieved in this case, especially for longer action sequences (Figures 6D,E).

With the *goal-directed exploration* method, actions are generated by trying to reach an internally (randomly) determined goal (i.e., reach a specific height with the ball) based on already acquired state-action-effect associations. As long as the model does not know how to reach this ball-related goal it moves the arm to random positions. As soon as the model moves into a state that is associated with the goal, the action selection mode changes. In 50% of the cases, it approaches the goal of throwing the ball to a specific height directly, consequently facilitating the generation of useful long action sequences. In the other 50%, the arm is moved to a random position, as before, to be able to explore alternative action sequences. The value of 50% yielded the best performance in the current task in pilot simulations. This method corresponds to a situation in which the child tries to throw the ball to different heights completely on its own, and without any previous knowledge of the task. Figures 6D,E show that this action generation method is slightly superior to random exploration.

The *best effect* method assumes that the child knows through which sequence of states it has to travel in order to reach the goal. This corresponds to a situation where the sequence of states may have been shown to the child by a teacher. The teacher is able to tell the child to which position to move the arm next, but of course it cannot tell the child which action nodes to activate. In 50% of cases, the best effect method attempts to reach the next state in the sequence by executing the best action currently known for reaching that next state; otherwise, it activates a random action node. Again, the value of 50% yielded maximal performance in pilot simulations. This method can be considered to provide the maximum information that could be realistically obtained. Even though performance is high, it is unable to reach all goals. Moreover, actions are sequenced sub-optimally in more than 10% of the cases (Figures 6D,E).

Finally, the *best action* method is of rather theoretic interest. It randomly selects a goal and then produces the optimal action sequence to reach it. This allows us to test if the model is able to store longer action sequences, given that only optimal throwing movements serve as learning examples. Figures 6D,E show that this method results in perfect performances. This shows that the model is structurally able to store state-action-effect associations that enable perfect behavior. This implies that the model’s performance is mostly limited by the necessarily suboptimal learning experience.

In conclusion, it seems reasonable to assume that natural behavior in learning a novel task is likely somewhat better than the goal-directed exploration method, but not as good as the best effect method. Thus, even a rather simple task with 12 different actions can only be partially mastered by the learning algorithm suggested by Ideomotor Theory.

#### Consolidation and planning

The previous section showed that the performance of our model of Ideomotor Theory depends critically on what actions are experienced during learning. Even if all the individual elements of an action sequence can be produced, they need to be executed in exact sequence during learning to be able to reproduce certain goals. Unfortunately, the probability that useful or even optimal action sequences are tried out during learning decreases exponentially with increasingly complex tasks.

While the basic state-action-effect triplets that constitute the elements of longer action chains can be easily acquired, learning entire sequences is difficult. This could be due to limitations in the information acquired during learning or the ineffective use of this information. To test the latter hypothesis, we tested whether reprocessing the acquired state-action-effect episodes could improve the performance of the model.

Two different modes of such reprocessing can be distinguished. First, the experiences of practice could be processed offline after learning. Indeed, it has been shown that performance in novel skills may increase after learning during times of rest (Brashers-Krug et al., 1996; Korman et al., 2007). This process is usually called consolidation.

Second, individual state-action-effect episodes may be sequenced before trying to reach specific goals, a process that might correspond to (motor) planning. In line with this reasoning is the finding that planning more complex or longer movements takes more time than planning simple movements (e.g., Rosenbaum et al., 1984; Munro et al., 2007). Note that if a consolidation mechanism, a planning mechanism, or both are necessary for successful learning this implies that associative learning alone is not sufficient and that effect anticipations do not always trigger actions directly.

For the sake of the example, we assume that the child practices 1000 ball throws a day. The consolidation mechanism of the model is invoked after daily practice and simulates another 1000 ball throws, based on the acquired state-action-effect links. This allows the formation of novel indirect state-action-effect links by learning from simulated action sequences that were not actually experienced.

The planning mechanism is invoked before generating actions in the test phase (but not for action generation in the acquisition phase). The planning mechanism systematically chains experienced state-action-effect links in order to reach remote goals. It is implemented by spreading activation repeatedly from the goal state or from states from which the goal can be reached to other states, thus creating new associations between actions and their indirect effects. This technique is known as dynamic programming (Bellman, 1957). Of course, both mechanisms could be realized in very different ways in the brain and we do not claim that our approach necessarily reflects these mechanisms in detail. The aim of the following simulations is to test whether consolidation or planning mechanisms can exploit the so-far learned state-action-effect associations more efficiently.

To evaluate the impact of consolidation and planning, we simulated the 12 action nodes ball-throwing example with an overall goal-directed training of 10.000 episodes. We used setups with and without planning or consolidation (100 simulation runs for each condition). Additionally, we simulated tasks with up to 96 nodes with and without consolidation and planning to see how these mechanisms affect performance in more complex tasks (20 simulation runs for each condition). During testing, each goal was pursued from each possible state 10 times.

**Figure 7:**
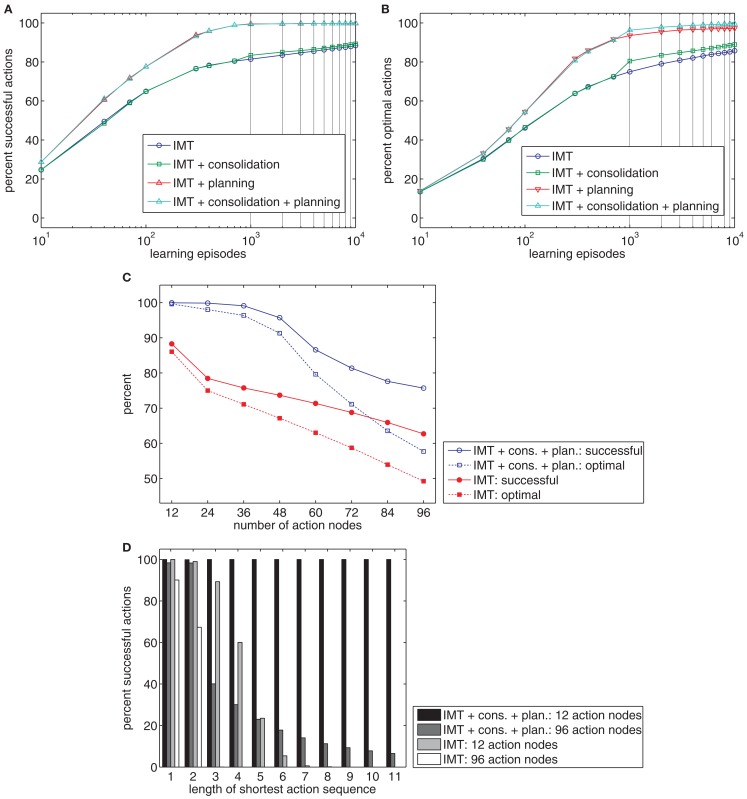
**(A,B)** The charts show the percentage of goals reached **(A)** or reached in the most efficient way **(B)** after different numbers of learning episodes. Four models were tested: pure Ideomotor Theory (IMT), an extension of IMT with a consolidation mechanism, an extension of IMT with a planning mechanism, and an extension of IMT with both kinds of mechanisms. **(C)** The chart shows the percentage of successful and optimal actions for learning tasks with different numbers of action nodes for the pure IMT model or models with consolidation and planning. **(D)** The chart shows the percentage of successful actions by the shortest action sequence length required to reach a goal.

Figure 7A shows that both planning and consolidation increase the percentage of goals reached. Whereas consolidation improves results only slightly, the planning mechanism yields a dramatic increase in performance. The success rate of about 90% after 10.000 trials of the models without planning is already surpassed after 200 trials if planning is employed. Whereas the overall success rate is little affected by the consolidation mechanism, Figure 7B shows that consolidation increases the percentage of optimally sequenced actions. This improvement is visible for models with and without planning. This suggests that planning and consolidation may play complementary roles. Whereas planning enables sequencing novel actions from acquired state-action-effect links, consolidation tends to improve the efficiency of these links.

Finally, we compare the pure Ideomotor Theory model against models that include consolidation and planning for up to 96 action nodes. Figure 7C shows that planning and consolidation enables the model to acquire much more complex tasks than would be possible without these mechanisms. Figure 7D charts model performance by the minimum length of action sequences required to reach a goal after 10.000 learning episodes. It can be seen that these additional mechanisms are pivotal to generate longer action sequences.

In sum, the planning mechanism especially enables effective sequencing of actions after comparatively little practice. Moreover, such a planning mechanism also allows adjusting action sequencing to situational constraints (Butz et al., 2007). For example, many throwing heights can be achieved from various initial positions of the hand. If some positions cannot be reached in a specific situation due to external obstacles or reduced mobility of the arm, for example, planning mechanisms might provide the flexibility to take such constraints into account.

To conclude, it seems plausible from a psychological and functional perspective that additional mechanisms play a crucial role for the acquisition and execution of goal-directed behavior. This implies that strong Ideomotor Theory and, in particular, that the claim that effect anticipations shall *directly* trigger actions, provides an insufficient account in more complex tasks.

## Discussion

In the previous section we developed a simple model of Ideomotor Theory. Following the example of a child learning to toss a ball, we extended our computational analysis beyond the learning challenges of common experiments on Ideomotor Theory. Given the simplicity of Ideomotor Theory, the model did rather well in many tasks. First, the model accounted for simple one-to-one mappings as used in many experimental setups. However, learning took considerably longer if many distinct actions and effects had to be considered, if the system was noisy, and if irrelevant actions possibilities shrouded the task-relevant actions. Nevertheless, the model was able to reach various goals after extended practice. Second, the model was capable of performing a task in which various actions or action sequences reached identical goals. Controlling such tasks is a non-trivial feature which cannot be accomplished by a range of learning mechanisms. However, this ability is impaired to some extent if action nodes have broad receptive fields. Third, the model was able to account for learning in dynamic environments if the delay between actions and their effects was small. A major challenge identified for Ideomotor Theory is the formation of links between actions and delayed effects. Likewise, while Ideomotor Theory can account for action sequencing in simpler tasks, the successful production of longer action sequences requires a rather lengthy acquisition phase. To conclude, our model suggests that Ideomotor Theory provides a good account for efficient unsupervised learning if (1) effects follow actions in close temporal proximity, (2) actions are simple movements that do not require intricate sequencing, and (3) the range of potentially relevant actions and effects is restricted. If these conditions are not met, implementations of Ideomotor Theory require extensive learning to approach reasonable performance.

Functionally, these preconditions could be said to limit the usefulness of Ideomotor Theory as a general mechanism for the acquisition of goal-directed behavior. However, these functional shortcomings support the model from a psychological perspective, because these limitations resemble those of humans in three ways. First, it has been shown that action-effect associations are only learned if both appear in a narrow time window (Elsner and Hommel, 2004). Second, skills in the “zone of proximal development,” which refers to capabilities that are just a little more complex than those already possessed by a learner, can be readily acquired (Vygotsky, 1978). In contrast, human beings have difficulties in acquiring skills that go far beyond their current capabilities. In our model, this effect was mimicked in Case 5, when skill learning required sequencing actions. Whereas the model can easily learn novel skills that require the sequencing a small number of familiar actions, it is far more difficult to learn skills which require long action sequences. Third, action and stimulus dimensions need to be constrained for humans to acquire novel skills. This was also evident in our model. Learning benefited if a teacher provided information on the to be executed task and if the number of potential actions and effects was constrained (Cases 2 and 5). This also parallels learning in humans. When children learn new skills, parents, older children, or other persons often support learning (Rogoff, 1998) by, for example, guiding attention (Zukow-Goldring and Arbib, 2007). However, in some cases skill acquisition cannot be supported from the outside or is only supported to a limited degree. In these cases, skill acquisition requires a lot of time. For example, infant reaching movements converge toward an adult-like level only after about 2 years (Konczak and Dichgans, 1997).

Thus, in sum, it can be argued that the identified functional limitations of Ideomotor Theory resemble those of humans. To nevertheless enable learning, humans try to constrain the space of relevant actions and stimuli. If this cannot be achieved, this type of human learning is a time-consuming process.

### Challenges for strong ideomotor theory

The various learning tasks could all be mastered to some extent. However, in some cases extensive learning experience was necessary to reach a high performance level. This suggests that the representational structure proposed by Ideomotor Theory, which was captured by our model, is adequate to control behavior. The major challenge stems from the difficulty to gather enough and good learning examples to fill the representational structure. While this was already evident from analyzing scenarios with an increasing number of action nodes, this problem was most dramatically exposed if a task required action sequencing. This reasoning is further supported by the fact that the exploration strategy during the acquisition phase had a very strong impact on performance. In the unrealistic case that perfect action sequences served as input for the ideomotor learning mechanism, the model learned to control actions optimally. However, if action selection during learning was less ideal, models of identical structure performed worse and were partially unable to sequence longer actions. Thus, even in the simple ball-throwing task in which the arm of the child could assume only 12 different postures, a random or goal-directed learning scheme results in mediocre performance, even after throwing the ball 10.000 times.

### Implications

Our results have two implications that relate directly to Ideomotor Theory. First, they shed light on the potential role of intentionality. Second, they relate to the validity of the direct-activation claim.

#### Intentional actions

It has recently been debated if an intentional action mode is necessary to enable action-effect learning. It has been argued that freely chosen, intentional actions (but not reactions) on a stimulus are associated to their effects (Herwig and Waszak, 2009). However, action-effect learning in a forced choice acquisition phase has also been reported (Pfister et al., 2011). While intentional and stimulus-based actions may be functionally distinct (Waszak et al., 2005), our model is mute to the differences between these two modes. Nonetheless, our analysis hints at another facet of intentionality. In our example of sequencing actions, we contrasted a random and a goal-directed exploration method. The goal-directed exploration method mimicked the behavior of a person who moved the arm in order to reach the overarching goal of throwing the ball to a specific height. Such an overarching goal was not present in the random exploration method. Thus, the goal-directed exploration method is intentional whereas the random exploration method is unintentional. This suggests that, at least in tasks that require the execution of more complex actions, intentionality might affect the way actions are executed during early phases of learning. Whereas an intentional mode produces actions that are fairly well suited for ideomotor learning, purely random exploration is less efficient. Indeed, it has been shown that participants learning a novel sensorimotor task switch to successively more goal-directed action modes after having explored basic action-effect relationships (Sailer et al., 2005). However, it is questionable to what extent this reasoning can be applied to experimental tasks that frequently require minimal action coordination.

#### Planning

Strong Ideomotor Theory implies that effect representations directly trigger actions. This claim is central to strong Ideomotor Theory as it distinguishes it from many of its competitors (Shin et al., 2010). Our model suggests a more differentiated view on this topic. The analysis revealed that our model is able to account for learning short action sequences. However, when longer action sequences had to be generated, only a planning mechanism enabled effective goal-directed behavior. Moreover, computational models of motor planning have shown that such mechanisms provide a flexibility that cannot be accomplished by direct action-effect mappings (Kawato et al., 1990; Rosenbaum et al., 1995; Cruse, 2003; Butz et al., 2007; Herbort et al., 2010). Thus, our analysis suggests that the direct-activation claim may be justified if the effects can be realized by executing simple actions. However, if it is necessary to sequence longer chains of actions, indirect planning mechanisms, which mediate between goals and actions, seem to be employed.

### Comparison of ideomotor theory with models of motor learning and control

The previous section has shown that action-effect learning and goal-directed behavior may not be as simple as predicated by Ideomotor Theory. Our critique focused mainly on Ideomotor Theory’s claim that effect anticipations *directly* trigger actions. Setting this aside, the other claims of Ideomotor Theory seem generally feasible from a computational perspective. To illustrate this point, we want to discuss the relationship between Ideomotor Theory and (computational) models of motor learning. We focus on motor learning because motor learning can be considered to be one of the computationally most complex learning problems that human beings face. In the following we make two arguments. First, we show that Ideomotor Theory is an effective way to address learning without prior knowledge of sensorimotor contingencies. For this reason, we discuss other models only with respect to the issue of learning a novel task (for reviews discussing such models in more detail see: Todorov, 2004; Butz et al., 2007, 2008). Second, we argue that the mechanisms suggested by Ideomotor Theory need to be complemented with other approaches to account for human behavior.

#### Bootstraping action-effect learning

Several recent computational models of motor learning and control share the assumption that goals are represented in terms of sensory effects with Ideomotor Theory. Moreover, the acquisition of action-effect associations is central to these models (e.g., Kawato, 1999; Butz et al., 2007). However, note that the direction of the associations is emphasized. In the motor literature, the term “forward model” refers to a set of action-effect links. The term “inverse model” is usually used to describe a set of associations between effects and actions, which are the focus of the following discussion.

The most basic learning scheme to associate effects with the actions that cause them is *direct inverse modeling* (Kuperstein, 1988; Jordan and Wolpert, 1999). According to direct inverse modeling, one-to-one effect-action associations are extracted from random movements. Each time an action is executed and an effect is observed, direct inverse modeling updates the corresponding effect-action mapping. The updating is based on the difference between the action that has actually been executed and the action with which the acquired effect-action associations would have tried to realize the actual effect. Thus, direct inverse modeling seems closely related to ideomotor learning, as learning is possible without an external error signal. The key difference is that direct inverse modeling updates action-effect associations with a supervised learning mechanisms, such as the delta rule, whereas Ideomotor Theory suggests an updating according to an unsupervised Hebbian-like rule.

While this difference seems rather technical, it has considerable impact on the capabilities of the learning mechanisms. If multiple actions result in identical effects, direct inverse modeling may fail (Jordan and Rumelhart, 1992; Jordan and Wolpert, 1999). Furthermore, as direct inverse modeling associates each effect with a single action, it is impossible to associate an effect with potentially multiple traces of various previous actions. Hence, this scheme cannot be applied to tasks in which actions unfold in time. While this limitation can be circumvented to some degree by reformulating the learning problem (Bullock et al., 1993), the mechanism is considered to be rather ineffective (Jordan and Rumelhart, 1992; Jordan and Wolpert, 1999) in dynamic settings with redundant action possibilities.

More advanced learning mechanisms do not suffer from the limitations of direct inverse modeling (e.g., distal supervised learning, Jordan and Rumelhart, 1992; feedback error learning, Kawato and Gomi, 1992). However, such mechanisms require an external error signal (for a discussion see Butz et al., 2007). This implies that there is some additional knowledge source available that provides information on how to improve one’s actions. Thus, these learning schemes may refine skills and improve performance but they cannot bootstrap action-effect learning. Moreover, these supervised learning schemes usually encode the single optimal action for each possible goal. This may be computationally efficient but may be disastrous if optimality criteria change. For example, an approximately straight movement path may be learned because it can be considered optimal for simple point-to-point movements (Flash and Hogan, 1985). However, straight movements are useless if there are obstacles in the way. Since other previously suboptimal actions are not encoded by the supervised learning schemes, alternative action sequences cannot be generated. In conclusion, supervised learning mechanisms have two limitations. First, action-effect learning in a novel situation is impossible. Second, behavior cannot be quickly adapted to changing task constraints.

Finally, Schmidt’s (1975) Schema Theory is a prominent framework of motor skill learning. According to Schema Theory, motor skills are organized around schemata that map goals onto suitable actions and the sensory input that usually accompanies their execution. A key feature of these schemata is their ability to parameterize actions (e.g., the strength of a ball throw) with respect to a goal (e.g., the target height of the ball) and initial conditions. Thus, the Schema approach offers an account for how a single skill can be applied to different tasks, such as throws of different height. While this account is highly attractive, Schema Theory has been formulated on a rather structural level. The precise learning mechanism that enables the abstraction of schemata from individual experiences or the generation of new schemata has not been formulated (Schmidt, 1975, 2003). Thus, Schema Theory does not offer a learning mechanism itself but is built on the assumption that such a mechanism exists. Whether learning mechanisms similar to those suggested by Ideomotor Theory could offer an implementation for schema generation has yet to be evaluated.

Thus, given the failure of direct inverse modeling for action-effect learning in redundant or dynamic tasks, the failure to bootstrap action-effect learning with supervised learning schemes, and the general inflexibility of both approaches, one can ask whether Ideomotor Theory can make a contribution. We think the answer should be “yes.” Our analysis has shown that ideomotor learning does not require an error signal or any prior information about the relationship between actions and effects. Moreover, ideomotor learning is able to cope with action redundancy, as has been shown by the paper plane example. Finally, ideomotor learning can handle situations in which actions and effects unfold in time. Thus, it seems that ideomotor learning could be a candidate to explain initial motor learning. Indeed, a simple computational model of Ideomotor Theory can account for learning to control a simple dynamic limb (Herbort et al., 2005). However, ideomotor learning can also lay the basis for more complex motor behavior. In our SURE_REACH model, we applied the principles of ideomotor learning to the control of a redundant arm (Butz et al., 2007; Herbort and Butz, 2007; Herbort et al., 2010). As in our last examples, the SURE_REACH model also deviates from pure Ideomotor Theory by including a planning mechanism. The model shows that ideomotor learning and a planning mechanism enables to explain highly adaptive behavior, such as the avoidance of obstacles, the reduction of the motion of injured joints, the integration of externally and internally defined constraints, and anticipatory adjustments of movements to subsequent actions. Thus, it can be concluded that the principles of ideomotor learning, as simple as they might be, can result in surprisingly adaptive and efficient behavior.

#### Complementary mechanisms

The previous section has shown that Ideomotor Theory, in contrast to many other theories, offers an effective unsupervised learning mechanism. In turn, future extensions of Ideomotor Theory could benefit considerably by adopting aspects of current models of motor learning and control.

Current models of motor learning and control distinguish clearly between predicting the consequence of an action (with forward models) and selecting an action to produce an effect (inverse models, Jordan and Rumelhart, 1992; Kawato and Gomi, 1992). This distinction is also made in Adams’ (1971) closed loop theory and Schmidt’s (1975) Schema Theory. The distinction is based on the findings that forward models are acquired faster than inverse models (Flanagan et al., 2003) and that both types of models assume different functions (Desmurget and Grafton, 2000). For example, in contrast to inverse models, which are primarily involved in control, forward models may help to cancel out noise, improve action selection by establishing an internal control loop, or even support inverse model learning (Karniel, 2002). In contrast to these considerations, Ideomotor Theorists describe the link between actions and effects as “bidirectional” (e.g., Elsner and Hommel, 2004; Shin et al., 2010), thereby neglecting potentially different mechanisms underlying mappings in different directions.

Moreover, in many models it is assumed that basic information is further refined during motor learning. One possible way to refine a motor skill is to use the output of the ideomotor model as a teaching signal for a secondary controller that improves performance and also mostly determines control signals (Kawato et al., 1987). In addition, basic acquired action-effect associations could be abstracted into schemata to form the basis of higher order motor skills (Schmidt, 1975). In this view, Ideomotor Theory could describe early processes of skill acquisition. It is likely to be complemented by other mechanisms later on.

Finally, the future extension of models of Ideomotor Theory should elaborate on mechanisms to model state- or context-dependent action-effect learning. In our Case 5 we introduced simple state-action-effect associations. However, it has been suggested that action-effect associations are only stored context-dependently if no contingent relationship between actions and effects can be otherwise established (Hoffmann, 2003). To accommodate such a process, it is likely that the representational structure of action nodes, effect nodes, and context nodes has to be adapted during learning. This, however, is out of the scope of our current model.

In conclusion, to model the acquisition of goal-directed behavior, Ideomotor Theory should be embraced as a core element. However, it needs to be integrated into larger frameworks to account for the control of actions, the refinement of movement skills, or abstraction processes.

## Summary and Conclusion

Ideomotor Theory is a framework that explains action-effect learning without prior knowledge with an astonishingly simple mechanism. Whereas the claim that actions are triggered by the anticipation of desired effects has found considerable empirical support, the assumptions that actions are directly associated to their effects during learning and that effect anticipation *directly* trigger actions have been examined rarely and indirectly. Here we took a computational approach to evaluate whether these assumptions are theoretically suited to explain goal-directed action control and action-effect learning. We developed a simple computational model of Ideomotor Theory and subjected it to a number of different learning tasks. In general, the model operated successfully on a wide range of tasks. Similar to humans, the model had difficulties if the range of potentially relevant sensory and action nodes was very large. Also similar to humans, the model failed to associate actions with delayed effects. However, when learning tasks require sequencing motor commands, which is the case for even simple reaching or grasping movements, Ideomotor Theory failed. This limitation mainly arose due to the model’s restriction that effect anticipations should directly trigger actions. When adding planning and possibly consolidation mechanisms and thus deviating from Ideomotor Theory’s claim that effect anticipations directly trigger actions, effective goal-directed behavior was achieved even for tasks where a proper sequencing of motor commands needs to be learned.

To conclude, from a computational point of view Ideomotor Theory offers a surprisingly sound basis to understand the acquisition of goal-directed behavior. However, the assumption that effect anticipations directly trigger actions can only be upheld for learning tasks that require the learning of a mapping from sensory to motor space. If actions unfold in time, additional planning mechanisms are inevitable.

## Conflict of Interest Statement

The authors declare that the research was conducted in the absence of any commercial or financial relationships that could be construed as a potential conflict of interest.

## Footnotes

^1^Actions are selected with equal probability and independent of action selections in previous learning episodes.

^2^In many real-world tasks this kind of noise can be controlled to some extent, e.g., by moving slowly (Fitts, 1954). Our exemplar task is too simple to offer the ability to model such strategic adjustments. However, it is conceivable that such strategic adjustments can be integrated in our model by including information about motor noise in effect representations.

^3^The activity of node *A_j_* when executing the *i*-th action was defined as the integral of the Gaussian probability density function with mean *i* between *j* − 0.5 and *j + *0.5.

^4^A ball thrown with the strongest action *A*_4_ reached the peak of its trajectory after 0.5 s, about 1.2 m above the hand (gravity constant of 9.81 ms^−2^). The ball then accelerated down again. The peak height of throws with other actions *A_i_* was proportional to the index *i* of the action: *A*_1_, *A*_2_, *A*_3_ produced heights of 0.3, 0.6, and 0.9 m, respectively. The four effect nodes covered the range from the height reached by the weakest throw *A*_1_ to the strongest throw *A*_4_ equidistantly. In each time step, the effect node encoding the height closest to the ball position was active.

^5^The following relationship between situations, actions, and effects were used. Let *n* be the number of different actions, *e*(*t* − 1) the state before the execution of the action *a*(*t*), *a*(*t*) the action, *e*(*t*) the effect after the execution of action *a*(*t*), and *t* be a specific point in time. Action *a*(*t*) was encoded by setting *A*(*t*)*_a(*t*)_* = 1 and all other nodes to *A*(*t*)*_i_* = 0, *i* ≠ *a*(*t*). Likewise, state *e*(*t*) was encoded by setting *E*(*t*)*_e(*t*)_* = 1 and all other nodes to *E*(*t*)*_i_* = 0, *i* ≠ *e*(*t*). To initialize an episode, time was set to *t* = 1, *e*(0) was either set to a random integer between 1 and *n* (learning) or to a specific value in that range (testing). In the case that *e*(*t* − 1) + 1 ≥ *a*(*t*), the effect was *e*(*t*) = *a*(*t*), i.e., ball and arm were moved together. Otherwise, the ball was thrown with *e*(*t*) = *n* − 1 + *a*(*t*) *−* *e*(*t* − 1) and the episode ended. During learning, actions *a*(*t*) were randomly set to a value between 1 and *n*. After each action, the state-action-effect weights *m* were updated, including a trace of past states and actions. For each time step *u* that has already passed in the episode (*u* = 1‥*t*), the following update rules were used: *m*_*e*(*u* − 1), *a*(*u*), *e*(*u*)_ = *m*_*e*(*u* − 1), *a*(*u*), *e*(*u*)_ + *f* (*t* − *u*). If there was no memory trace, *f* (*x*) was set to 1.0 when *x* was 0.0, and otherwise to zero. If the exponential function was used, *f* (*x*) = *e*^−*x*^. If a flat function was used, *f* (*x*) = 1. During testing, actions were generated by the weights associated to the state, each potential action *j*, and the desired effect *e**, *a*(*t*)*_j_* = *m*_*e*(*t*),*j,e**_. The action associated to the highest weight was then executed. If several actions had maximum weights, a random choice was made. An episode ended if the ball was thrown, if the desired effect was produced, or if more than twice as many steps elapsed as would be necessary to be able to reach either effect from either initial state. The evaluation was conducted 100 times for each initial state and each desired effect.

^6^State-action-effects weights at time t after action execution were increased by *e*^−0.2t^.

^7^State-action-effects weights at time t after action execution were increased by *e*^−0.2t^.
